# Carbon and hydrogen stable isotope fractionation due to monooxygenation of short-chain alkanes by butane monooxygenase of *Thauera butanivorans* Bu-B1211

**DOI:** 10.3389/fmicb.2023.1250308

**Published:** 2023-09-25

**Authors:** Carsten Vogt, Zhiyong Song, Hans-Hermann Richnow, Florin Musat

**Affiliations:** ^1^Department of Isotope Biogeochemistry, Helmholtz Centre for Environmental Research–UFZ, Leipzig, Germany; ^2^Section for Microbiology, Department of Biology, Aarhus University, Aarhus, Denmark; ^3^Department of Molecular Biology and Biotechnology, Faculty of Biology and Geology, Babeş-Bolyai University, Cluj-Napoca, Romania

**Keywords:** alkanes, CSIA, monooxygenase, *Thauera butanivorans*, stable isotopes, isotope fractionation

## Abstract

Multi element compound-specific stable isotope analysis (ME-CSIA) is a tool to assess (bio)chemical reactions of molecules in the environment based on their isotopic fingerprints. To that effect, ME-CSIA concepts are initially developed with laboratory model experiments to determine the isotope fractionation factors specific for distinct (bio)chemical reactions. Here, we determined for the first time the carbon and hydrogen isotope fractionation factors for the monooxygenation of the short-chain alkanes ethane, propane, and butane. As model organism we used *Thauera butanivorans* strain Bu-B1211 which employs a non-haem iron monooxygenase (butane monooxygenase) to activate alkanes. Monooxygenation of alkanes was associated with strong carbon and hydrogen isotope effects: ε_bulk_C = −2.95 ± 0.5 ‰ for ethane, −2.68 ± 0.1 ‰ for propane, −1.19 ± 0.18 ‰ for butane; ε_bulk_H = −56.3 ± 15 ‰ for ethane, −40.5 ± 2.3 ‰ for propane, −14.6 ± 3.6 ‰ for butane. This resulted in lambda (Λ ≈ *ε*H_bulk_/*ε*C_bulk_) values of 16.2 ± 3.7 for ethane, 13.2 ± 0.7 for propane, and 11.4 ± 2.8 for butane. The results show that ME-CSIA can be used to track the occurrence and impact of monooxygenase-dependent aerobic processes converting short-chain alkanes in natural settings like marine and terrestrial seeps, gas reservoirs, and other geological formations impacted by natural gas.

## 1. Introduction

Alkanes are the major fraction of non-degraded crude oils, accounting for over 50% by mass (Tissot and Welte 1984). Short-chain, gaseous alkanes are mainly released in the biosphere from deep-seated oil or gas reservoirs by natural seepage or during the anthropogenic exploration and exploitation of fossil fuels; global annual emissions have been estimated at 42–64 Tg/year for methane, about 10 Tg/year for ethane, propane and n-butane, respectively, and about 4 Tg/year for isobutane (Etiope and Ciccioli 2009; Pozzer et al., 2010; Musat et al., 2016).

Owing to their long-time presence in the biosphere, short-chain alkanes are used as growth substrates by a wide diversity of aerobic and anaerobic microorganisms which have evolved different alkane oxidation pathways. Under anoxic conditions, biodegradation of gaseous non-methane alkanes has been observed with sulfate and nitrate as terminal electron acceptors. Two major functionalization mechanisms and subsequent degradation pathways can be distinguished. Nitrate- and sulfate-reducing bacteria use glycyl radical enzymes which catalyze the hemolytic cleavage of a C-H-bond of the alkane, followed by carbon–carbon addition of the generated alkyl radical to fumarate (Savage et al., 2010; Musat 2015; Musat et al., 2016; Wu et al., 2022); the reaction can take place simultaneously at the terminal and subterminal carbon atoms (propane) or only at the subterminal carbon atom (butane) (Kniemeyer et al., 2007; Jaekel et al., 2014). The formed alkylsuccinates are subsequently channeled into the central metabolic pathways by ligation to coenzyme A, C-skeleton rearrangement, and beta-oxidation (Musat 2015; Chen et al., 2022). Distinct mechanisms of activation and downstream oxidation pathways have been recently documented for short-chain alkane oxidizing archaea forming syntrophic associations with partner sulfate-reducing bacteria. This pathway proceeds analogously to the anaerobic oxidation of methane and is initiated by enzymes bearing similarities to methyl-coenzyme *M* reductases (MCR), named alkyl-coenzyme *M* reductases, or ACR. This leads to formation of alkyl-coenzyme *M* as alkane activation products, which are further converted to acyl-CoA by yet unknown reactions, followed by beta oxidation and channeling of the generated acetyl-CoA into the C1 pathway, enetually leading to complete oxidation to carbon dioxide (Laso-Perez et al., 2016; Chen et al., 2019; Hahn et al., 2020; Zehnle et al., 2023).

In oxic settings, short-chain alkanes are oxidized by a high diversity of aerobic microorganisms (Shennan 2006) which could exert a tremendous impact on geochemical cycles in environments impacted by massive emissions of hydrocarbons (Valentine et al., 2010). Despite their phylogenetic diversity, aerobic microorganisms use common mechanistic principles to activate the alkane molecules. This is done by hydroxylation of the terminal (primary) carbon atom of the alkane, yielding primary alcohols; in addition, propane and butane are activated to a lesser extent at their subterminal (secondary) carbon atoms, yielding secondary alcohols. Both reactions are catalyzed by molecular oxygen-dependent alkane hydroxylases, also named monooxygenases. Various structurally different types of alkane hydroxylases are currently known, including methane monooxygenases, alkane hydroxylases related to an integral-membrane non-haem diiron monooxygenase (AlkB), or alkane hydroxylases related to cytochrome P450 (Rojo 2009). A well-studied strain assimilating gaseous alkanes is *Thauera butanivorans* (formerly *Pseudomonas butanovora*, Dubbels et al., 2009). It activates gaseous alkanes primarily by monooxygenation of the primary carbon atom using a butane monooxygenase, a non-haem iron monooxygenase related to soluble methane monooxygenases (Rojo 2009).

In the last two decades, compound-specific stable isotope analysis (CSIA) has been established as state-of-the-art technique for source appointment and for monitoring of (bio)degradation reactions at contaminated sites (Elsner 2010; Fischer et al., 2016). The principle of CSIA is that isotopologues react in slightly different rates upon rate-limiting steps of (bio)chemical reactions; in most cases the lighter isotopologues react faster, leading to residual substrate pools enriched in heavier isotopes, and to reaction products pools enriched in lighter isotopes. This is termed normal kinetic isotope fractionation. The kinetic isotope fractionation effect is dependent on the rate limitation of bond change in the irreversible bond cleavage reaction (commitment to catalysis) and is related to the mode of bond change. The extent of observed isotope fractionation can be affected by rate limitation prior to the bond change reaction which is typically lowering the observed isotope fractionation – this is termed masking of the kinetic isotope effect (KIE). The magnitude of isotope fractionation, defined by the enrichment factor *ε*, depends on various parameters, like the type of (bio)chemical reaction (see above), the mass of the respective isotopes, or bioavailability restrictions and other factors such as uptake of reactant into the cell, transport of reactant to the enzyme, or binding to the enzyme (Vogt et al., 2016). The latter leads to masking of isotope fractionation which can be circumvented by analyzing stable isotopes of two or more elements within the molecule (Multi element-CSIA, ME-CSIA) which may cancel out the rate limitation prior the commitment of catalysis. The fractionation of stable isotopes of two (or more) elements usually correlates over a wide concentration range, so that the resulting correlation factor (termed lambda, Λ) can be used to specify a distinct (bio)chemical reaction. Hence, in case of hydrocarbons, analysis of isotope fractionation of both stable carbon and hydrogen isotopes allows characterizing distinct (bio)chemical hydrocarbon activation mechanisms. Whereas Λ values and isotope fractionation factors of various aerobic and anaerobic activation mechanisms for aromatic hydrocarbons have been determined (for an overview see Vogt et al., 2016), less data is available for similar bond cleavage reactions initiating a degradation pathway of alkanes. Jaekel et al. (2014) characterized the reaction of propane and butane activation by addition to fumarate by ME-CSIA using sulfate-reducing enriched and pure cultures. Due to the gaseous nature of the short-chain alkanes, carbon and hydrogen stable isotope fractionation can be significantly impacted by limited alkane diffusion toward the cells in systems with gas and liquid phases. It has been shown that mass transfer limitations caused by insufficient mixing, low substrate bioavailability, or high cell densities as those occurring in aggregates and biofilms, lead to substantial decrease of observable isotope fractionation (Templeton et al., 2006; Staal et al., 2007; Kampara et al., 2008, 2009; Jaekel et al., 2014), emphasizing the importance of ME-CSIA to characterize the reaction patterns. In addition, the anaerobic oxidation of propane via the addition to fumarate mechanism was characterized by intramolecular, position-specific carbon isotope fractionation, which allowed to detect and quantify anaerobic oxidation processes in natural gas reservoirs (Gilbert et al., 2019). Although C and H isotope fractionation associated with the aerobic oxidation of short-chain alkanes have been reported for sediment incubations (Kinnaman et al., 2007; Bouchard et al., 2008), a characterization of the stable isotope fractionation of aerobic monooxygenation using defined cultures is currently missing. In this study, we determined for the first-time bulk and reactive position-specific enrichment factors for carbon (ε_C_) and hydrogen (ε_H_) stable isotopes and bulk Λ values for the monooxygenation of ethane, propane and butane, using as model organism *Thauera butanivorans* Bu-B1211, a strain which contains a single alkane monooxygenase dubbed butane monooxygenase. We further compare the isotope fractionation factors of monooxygenation with fractionation factors obtained for anaerobic degradation pathways. The data will lead to a better understanding of isotope effects linked to the biodegradation of gaseous alkanes in the environment.

## 2. Materials and methods

### 2.1. Chemicals

Ethane, propane, and butane (purity ≥ 99.95 mol %) were purchased from Air Liquide (Düsseldorf, Germany).

### 2.2. Culture and cultivation conditions

*Thauera butanivorans* strain Bu-B1211 (DSM 2080) was obtained from the Leibnitz Institute DSMZ German Collection of Microorganisms and Cell Cultures, Braunschweig, Germany. Strain Bu-B1211 was cultivated in aerobic, bicarbonate-buffered mineral salt medium provided with gaseous alkanes as sole source of carbon and energy. The medium contained 0.5 g KH_2_PO_4_, 0.3 g NH_4_C1, 0.4 g MgSO_4_·7H_2_O, 0.1 g CaCl_2_·2H_2_O, and 1.0 g NaCl in 1 L of distilled water. After autoclaving and cooling under air in bottles sealed with butyl rubber stoppers, the following substances were added (according to Widdel and Bak 1992): CO_2_ (10%, v/v), 30 mL NaHCO_3_ solution (84 g/L, autoclaved under CO_2_), vitamins, EDTA-chelated mixture of trace elements, and selenite and tungstate solution. The pH of the medium was adjusted to 7.0–7.4.

*Thauera butanivorans* was cultured in 120 mL serum bottles containing 30 mL medium under a headspace of air (21% v/v O_2_). Ethane, propane, and butane were added to the headspace at 1 bar partial pressure (final pressure: 2 bar). The bottles were closed with butyl rubber stoppers. Subcultures were prepared by inoculating fresh culture medium with 3% v/v of an active culture. The cultures were incubated on a horizontal shaker (100 rotations per minute, rpm) at 28°C in a horizontal position in order to increase the surface-to-volume ratio and thus the rate of mass transfer of alkanes into the liquid medium, due to their low solubility in water.

### 2.3. Isotope fractionation experiments

Isotope fractionation experiments with ethane, propane and butane were done in 120 mL serum bottles containing 30 mL medium and defined amounts of pure ethane (5 mL), propane (3.6 mL) or butane (2.8 mL). In order to stimulate the biodegradation of ethane, the medium was supplemented with FeSO_4_·7H_2_O and yeast extract, in final concentrations of 0.1 g/L, respectively. The bottles were subsequently inoculated with 1 mL of culture previously grown on the same gaseous alkane. For each alkane, 15 parallel bottles were prepared, of which 3 bottles served as sterile controls without added cells. The microcosms were incubated at 28°C on a rotary shaker (100 rpm) and monitored with respect to alkane degradation and growth, the latter measured as changes in optical density (OD 600 nm). After approximately 0, 10, 20, 50, 60, 80, 90, and 95% of alkane had been consumed, individual cultures were inactivated by the addition of 4 M NaOH (1 mL), adjusting the pH to 12, and subsequent heating in a water bath at 80°C for 15 min. Inactivated cultures were stored at room temperature until analysis. The sterile, control bottles were treated by the same procedure at the times when the biodegradation had reached 0, 50 and 95% in inoculated cultures, respectively.

### 2.4. Analytical methods

Alkane concentrations were quantified using a gas chromatograph (Chrompack CP-3800, Varian) equipped with a flame ionization detector. The alkanes were resolved using a GS-Q PLOT column (30 m × 0.53 mm, 30 μm film thickness; Agilent Technologies), with N_2_ as carrier gas at a flow rate of 3 mL min^−1^. The oven was maintained at 140°C, with the injection and detection temperatures maintained at 220°C and 350°C, respectively. Ethane, propane, and butane concentrations were calculated based on external calibration curves using the pure gases as standards and reported as the mean values of duplicate measurements (technical replicates). Headspace samples (500 μL) were taken from the culture bottles using gastight glass syringes and injected with a split ratio of 1:50. For each culture and time point duplicate headspace samples were analyzed to account for possible instrumental deviations.

Carbon isotope analyses were determined by gas chromatography isotope-ratio mass spectrometry (GC-IRMS) using a GC (6,890 N Gas chromatograph, Agilent Technology) equipped with a PoraBOND Q column (50 m × 0.32 mm × 5 μm, Agilent Technology). The GC was linked via a GCCIII combustion unit (Thermo Fisher Scientific) and a ConFlo IV interface (Thermo Fisher Scientific) to an isotope ratio mass spectrometer (MAT 253, Thermo Fisher Scientific). For hydrogen isotope analyses a GC (7890A Gas chromatograph, Agilent technology) equipped with the same column as described for carbon isotope analyses was used. The GC was linked via a GC-Isolink (Thermo Fisher Scientific) and a ConFlo IV interface (ThermoFisher Scientific) to an isotope ratio mass spectrometer (MAT 253, Thermo Fisher Scientific). For both analyses He was used as a carrier gas, and the GC oven was maintained at 100°C for ethane, 140°C for propane and 180°C for butane, respectively. Samples were taken from the head space in volumes ranging from 50 to 1,000 μL and injected in split mode with a split ratio ranging from 1:20 to 1:3 into the split/splitless injector, maintained at 250°C. Each sample was measured in triplicate. The total analytical uncertainty with respect to both accuracy and reproducibility, estimated from the triplicate measurements, was always better than ±0.5‰ for δ^13^C and ± 10‰ for δ^2^H, respectively. The obtained isotope ratios were expressed in the δ-notation (δ^13^C and δ^2^H) relative to international isotope standards of the international atomic energy agency (Coplen 2011): Vienna Pee Dee Belemnite (VPDB) for stable carbon isotopes and Vienna standard Mean Ocean Water (VSMOW) for hydrogen isotopes. Pure ethane, propane, and butane gases were used as standards for the isotopic ratios before biodegradation.

Stable isotope fractionation of alkane hydroxylation was described by calculating bulk isotope enrichment factors (ε_bulk_) using the logarithmic form of the Rayleigh equation (Equation 1).


(1) $$\ln\left(\frac{\delta_{t}+1}{\delta_{0}+1}\right)=\varepsilon ln\left(\frac{C_{t}}{C_{0}}\right)$$

The isotope enrichment factors of the reactive position (ε_rp_), apparent kinetic isotope effects (AKIEs) and *Λ*-values were calculated as reported elsewhere (Jaekel et al., 2014).

In brief, bulk enrichment factors, ε_bulk_ (corresponding to ε in Equation 1) were converted to reactive position enrichment factors (ε_rp_) using Equation 2.


(2) $$\ln\left(\frac{n/x\cdot \delta_{t}+1}{\delta_{0}+1}\right)=\varepsilon_{rp}\ln\left(\frac{C_{t}}{C_{0}}\right)$$

Due to the hydroxylation at the C1 position of alkanes by butane monooxygenase, we used the following numbers for n (number of atoms in the molecule) and x (number of atoms in reactive positions, indicated in the following in boldface): ethane, *n* = 2 and *x* = 2 for carbon (H_3_**C**-**C**H_3_) and *n* = 6 and *x* = 6 for hydrogen (**H_3_**C-C**H_3_**); propane, *n* = 3 and *x* = 2 for carbon (H_3_**C**-CH_2_-**C**H_3_) and *n* = 8 and *x* = 6 for hydrogen (**H_3_**C-CH_2_-C**H_3_**); butane, *n* = 4 and *x* = 2 for carbon (H_3_**C**-CH_2_-CH_2_-**C**H_3_) and *n* = 10 and *x* = 6 for hydrogen (**H_3_**C-CH_2_-CH_2_-C**H_3_**). The errors of the obtained bulk and reactive position specific enrichment factors were given as a 95% confidence interval (CI) and were calculated by regression analysis as described elsewhere (Elsner et al., 2007).

AKIEs for carbon and hydrogen were calculated according to Equation 3.


(3) $$\mathrm{AKIE}=\left(\frac{1}{1+z\cdot \varepsilon_{rp}}\right)$$

Where *z* is the number of atoms in identical positions. We set *z* = 2 for C and 6 for H for ethane, propane, and butane.

Lambda (Λ) values were calculated according to Equation 4.


(4) $$\Lambda =\frac{\Delta \delta^{2}H}{\Delta \delta^{13}C}\approx \frac{\varepsilon_{H}}{\varepsilon_{C}}$$

## 3. Results and discussion

### 3.1. Consumption of alkanes by *Thauera butanivorans* and associated isotope fractionation

Under the cultivation conditions employed, *T. butanivorans* consumed over 80% of the added ethane, propane, and butane (initial concentrations of ~2.3, 1.6 and 1.3 mM, respectively) within around 40 h of incubation (Supplementary Figure S1). Alkane consumption was coupled to cell growth (Supplementary Figure S1; Takahashi et al., 1980). Within 60 h of incubation, over 95% of the initial amounts of propane and butane were consumed. Overall, the oxidation rates decreased when over 80% of the initially added alkane was consumed (Supplementary Figure S1), most likely a consequence of mass transfer limitations under low alkane concentrations in the gas phase. Notably, within the same incubation time, only 85% of the initially added ethane was consumed, suggesting that the monooxygenase initiating alkane oxidation has lower affinity for ethane than for the other alkanes.

**Figure 1:**
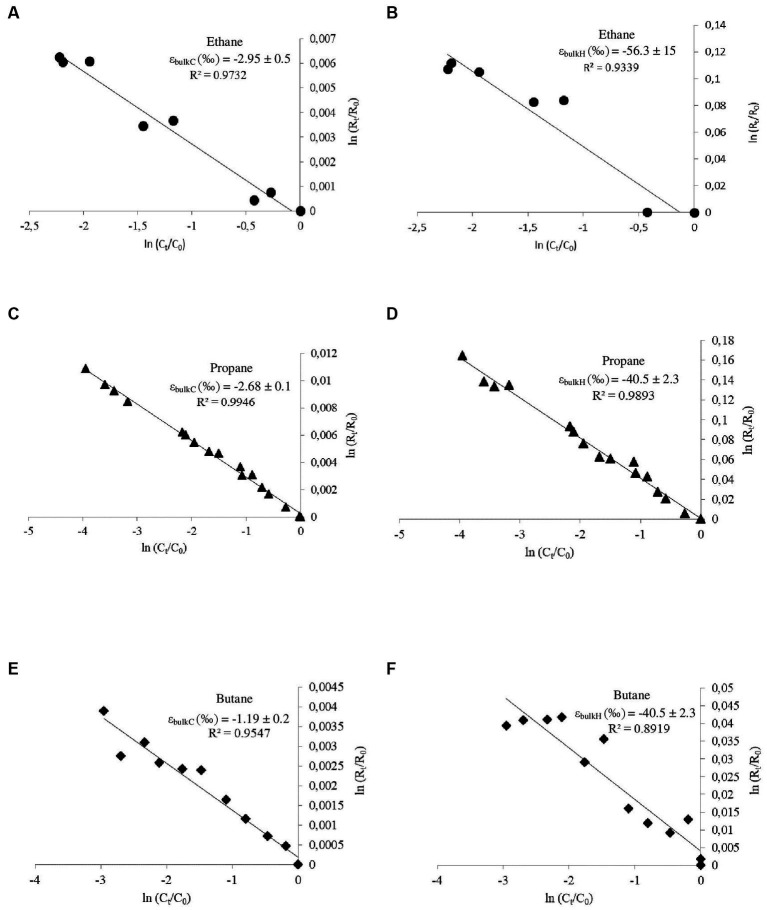
Double logarithmic plots of the isotopic composition versus the residual concentration of ethane **(A,B)**, propane **(C,D),** and butane **(E,F)** during monooxygenation by *T. butanivorans*; the lines correspond to a linear regression model, resulting in carbon **(A,C,E)** and hydrogen **(B,D,F)** enrichment factors (see Table 1, ε_bulkC_ and ε_bulkH_ values).

**Figure 2:**
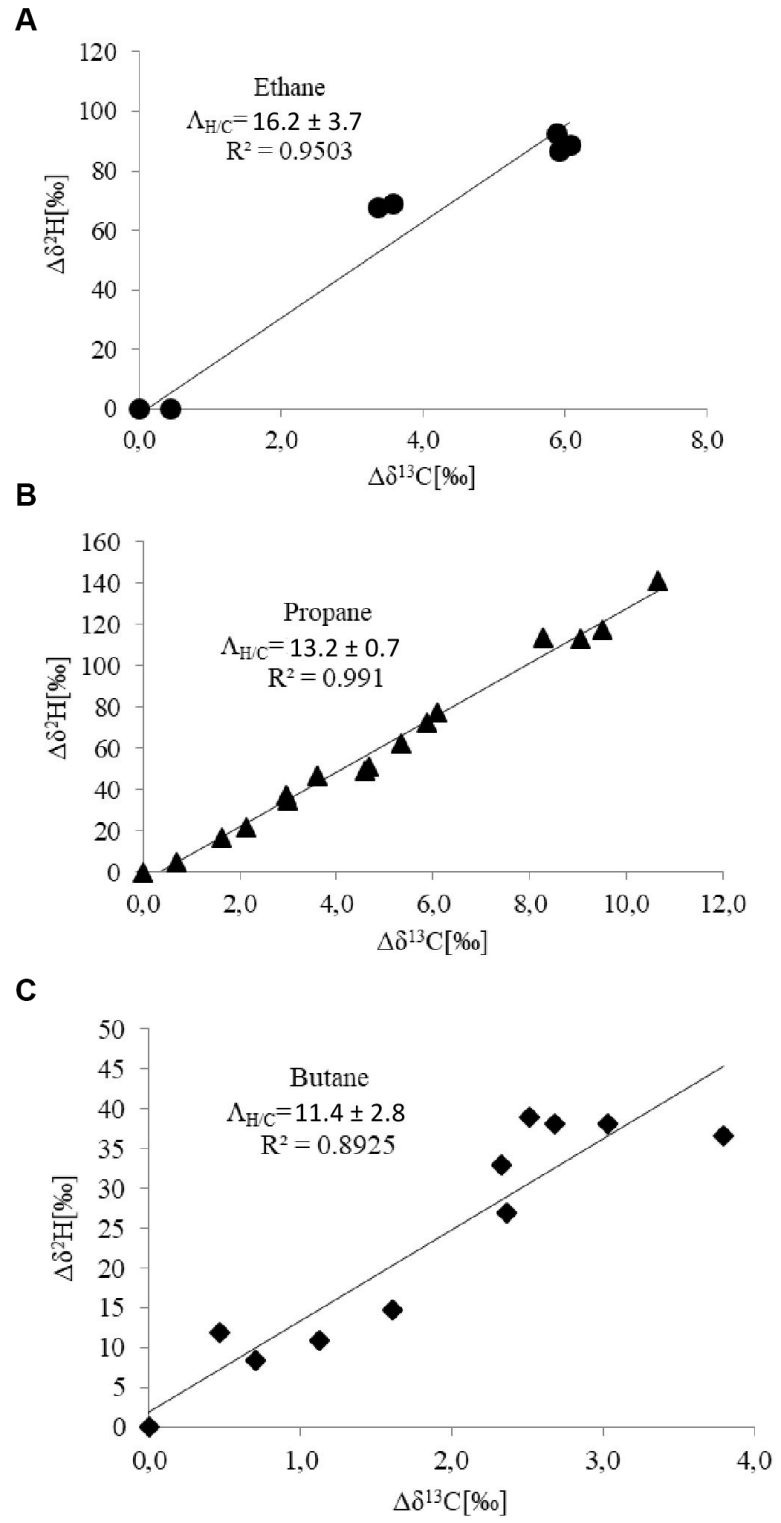
Plots of Δδ^2^H vs. Δδ^13^C for ethane **(A)**, propane **(B),** and butane **(C)** as substrates. The slopes of the regression curve give the Λ_H/C_ values.

Natural abundance stable isotope fractionation during consumption of a certain substrate is typically governed by the enzyme initiating the oxidation pathway (Elsner 2010; Vogt et al., 2016). Degradation of ethane, propane, and butane in *T. butanivorans* is initiated by the same enzyme, butane monooxygenase (sBMO), a soluble three-component diiron monooxygenase system with a broad substrate spectrum (Sluis et al., 2002; Dubbels et al., 2007). Experiments with purified sBMO showed that ethane is converted to ethanol, while, propane and butane are converted mainly to their corresponding primary alcohols (1-propanol and 1-butanol, respectively), which account for over 80% of the enzyme product (Dubbels et al., 2007); the rest of the enzyme products (about 20%) are the corresponding secondary alcohols, 2-propanol and 2-butanol. The pathway of 1-butanol assimilation was elucidated by Arp (1999): 1-butanol is further oxidized via butyraldehyde to butyrate, which is metabolized to acetyl-CoA by beta-oxidation. Mechanistically, like other soluble diiron monooxygenases, the catalytic cycle of sBMO is initiated by activation of molecular oxygen to a bound peroxide state, which homolytically cleaves the covalent C-H bond of the alkane. This generates an enzyme-bound alkyl radical and an iron-coordinated hydroxyl radical which recombine (rebound mechanism) to generate the hydroxylated alkane (an alcohol) (Widdel and Musat 2019). The oxidation of ethane, propane, and butane by *T. butanivorans* was associated with normal carbon and hydrogen isotope fractionation, hence ^13^C and ^2^H became enriched in the residual substrates during biodegradation (Figures 1A–F). This indicates that the cleavage of the C-H-bond during monooxygenation by sBMO is a rate-determining step of the enzymatic reaction. The correlation between substrate concentration and isotope ratios (*R*^2^) was always higher than 0.89 (Figures 1A–F), showing that the process could be described by the Rayleigh equation. Removal of alkanes was solely biotic, since no losses of ethane, propane or butane or changes in carbon and hydrogen isotope ratios were observed in abiotic controls (data not shown). Bulk and reactive-position-specific values for ε_C_ and ε_H_ as well as values for AKIE_C_ and AKIE_H_ are listed in Table 1. For both carbon and hydrogen isotopes, ε_bulk_ values decreased with increasing chain length: ε_bulk_C values of ethane, propane and butane were − 2.95 ‰, −2.68 ‰, and − 1.19 ‰, respectively, whereas ε_bulk_H values were − 56.3 ‰, −40.5 ‰, and − 14.6 ‰, respectively. Such trend is thought to be caused by dilution of isotope fractionation by additional non-reacting carbon and hydrogen atoms in longer alkanes. The same trend – decreasing enrichment factors at increasing chain length - was also observed for ε_rp_H values, which could be explained as a contribution of the additional enzymatic activation of propane and butane at the secondary C atoms (and thus the bounded hydrogen atoms) as described by Dubbels et al. (2007) for the butane monooxygenase of *T. butanivorans*, leading to a dilution of the isotope effect at the reactive position. In contrast, ε_rp_C values were in a similar range (Table 1). Since hydrogen isotope fractionation was around 10 times higher than carbon isotope fractionation, as expected for kinetic isotope effects associated with the cleavage of a C-H-bond, the dilution effect was probably below the detection limit for reactive carbon positions. AKIE values for C and H were 1.005–1.008 and 1.17–1.51, respectively. Lambda (Λ) values were rather similar for these three alkanes, with ethane showing the highest value (16.2 ± 3.7) and butane the lowest (11.4 ± 2.8) (Figure 2; Table 1). In summary, the isotope fractionation data are consistent with the hypothesis that the reaction mechanism of the butane monooxygenase of *T. butanivorans* is similar for all substrates.

**Table 1:** Enrichment factors (ε_bulkC_, ε_bulkH_), lambda (*Λ*) values and apparent kinetic isotope effects (AKIE_C_, AKIE_H_) for *n*-alkane activation by aerobic and anaerobic microbial strains or environmental samples.

**Culture/sample**	**Mixing conditions**	**Biochemical reaction**	**Substrate**							**Reference**
			**Ethane**							
			ε_bulk_C (‰)	ε_bulk_H (‰)	ε_rp_C (‰)	ε_rp_H (‰)	Λ	AKIE_C_	AKIE_H_	
*T. butanivorans*	Continuous	Monooxygenation	−2.95 ± 0.5	−56.3 ± 15	−2.95 ± 0.5	−56.3 ± 15	16.2 ± 3.7	1.006 ± 0.001	1.51 ± 0.21	This study
Marine sediments, aerobic degradation	NA	ND	−8.0 ± 1.7 ^6^	−61.9 ± 8.3			7.7	1.016 ^4,8^	1.59 ^4,8^	Kinnaman et al. (2007)
			**Propane**							
			ε_bulk_C (‰)	ε_bulk_H (‰)	ε_rp_C (‰)	ε_rp_H (‰)	Λ	AKIE_C_	AKIE_H_	
*T. butanivorans*	Continuous	Monooxygenation	−2.68 ± 0.1	−40.5 ± 2.3	−3.9 ± 0.2	−42.4 ± 2.4	13.2 ± 0.7	1.008 ± 0.0003	1.479 ± 0.04	This study
Marine sediments, aerobic degradation	NA	ND	−4.8 ± 0.9^6^	−15.1 ± 1.9			3.1 ^7^	1.015 ^4,8^	1.137 ^4,8^	Kinnaman et al. (2007)
Soil microcosms, aerobic degradation	NA	ND	−10.8 ± 0.7	ND				1.034 ^4,8^		Bouchard et al. (2008)
Strain BuS5	None	Fumarate addition	−2.6 ± 0.6	−16 ± 4	−7.9 ± 1.7	−61 ± 16	6.3 ± 0.9	1.008 ± 0.002	1.138 ± 0.04	Jaekel et al. (2014)
	Discontinuous		−5.2	ND						Kniemeyer et al. (2007)
	Continuous		−8.7 ± 1.1	−92 ± 6	−25.3 ± 3.3	−267 ± 31	11.9 ± 0.2	1.026 ± 0.003	2.143 ± 0.29	Jaekel et al. (2014)
Prop12-GMe^1^	None	Fumarate addition	−3.1 ± 0.4	−24 ± 4	−9.3 ± 1.1	−89 ± 14	7.9 ± 0.6	1.009 ± 0.001	1.217 ± 0.04	Jaekel et al. (2014)
	Discontinuous		−5.9							Kniemeyer et al. (2007)
	Continuous		−3.7 ± 0.5	−36 ± 4	−11.0 ± 1.6	−130 ± 18	10.0 ± 0.4	1.011 ± 0.002	1.352 ± 0.07	Jaekel et al. (2014)
Propane60-GuB	Discontinuous	Fumarate addition	−5.9	ND						Kniemeyer et al. (2007)
Marine sediments, anaerobic degradation	NA	ND	−4.8 ± 3.1 ^2^	−43.3 ± 5.1 ^2^			9.0 ^3^	1.015 ^4,5^	1.53 ^4,5^	Mastalerz et al. (2009)
			***n*-Butane**							
			ε_bulk_C (‰)	ε_bulk_H (‰)	ε_rp_C (‰)	ε_rp_H (‰)	*Λ*	AKIE_C_	AKIE_H_	
*T. butanivorans*	Continuous	Monooxygenation	−1.19 ± 0.18	−14.6 ± 3.6	−2.32 ± 0.36	−21.9 ± 5.4	11.4 ± 2.8	1.005 ± 0.001	1.171 ± 0.04	This study
Marine sediments, aerobic degradation	NA	ND	−2.9 ± 0.9^4^	ND				1.009 ^4,8^		Kinnaman et al. (2007)
Soil microcosms, aerobic degradation	NA	ND	−5.6 ± 0.1	ND				1.023 ^4,8^		Bouchard et al. (2008)
Strain BuS5	None	Fumarate addition	−1.8 ± 0.3	−9 ± 2	−3.5 ± 0.6	−21 ± 5	4.9 ± 1.2	1.007 ± 0.001	1.093 ± 0.03	Jaekel et al. (2014)
	Discontinuous		−1.6							Kniemeyer et al. (2007)
	Continuous		−5.0 ± 0.7	−32 ± 10	−9.9 ± 1.4	−74 ± 24	6.9 ± 1.2	1.02 ± 0.003	1.423 ± 0.2	Jaekel et al. (2014)
But12-GMe	None	Fumarate addition	−2.0 ± 0.5	−11 ± 4	−4.0 ± 1.1	−28 ± 9	5.9 ± 0.9	1.008 ± 0.002	1.127 ± 0.05	Jaekel et al. (2014)
	Discontinuous		−1.6							Kniemeyer et al. (2007)
	Continuous		−5.6 ± 0.5	−47 ± 5	−11.1 ± 1.1	−110 ± 11	8.7 ± 0.4	1.023 ± 0.002	1.78 ± 0.13	Jaekel et al. (2014)
But12-HyR^1^	None	Fumarate addition	−0.8 ± 0.3	−5 ± 2	−1.5 ± 0.7	−12 ± 5	5.3 ± 2.4	1.003 ± 0.001	1.05 ± 0.02	Jaekel et al. (2014)
	Continuous		−1.5 ± 0.4	−12 ± 2	−3.0 ± 0.7	−29 ± 1	7.7 ± 0.8	1.006 ± 0.002	1.129 ± 0.03	Jaekel et al. (2014)
Marine sediments, anaerobic degradation	NA	ND	−0.7 ± 0.4^9^	ND				1.003 ^4,5^		Mastalerz et al. (2009)

NA, not applicable; ND, not determined.

^1^Enrichment cultures forming large, dense aggregate.

^2^Mean values; the ε_bulk_C fractionation factors determined ranged from − 2.3 to − 10.2‰, and ε_bulk_H from − 39.5 to − 47‰.

^3^Calculated using the average values reported by Mastalerz et al. (2009).

^4^Calculated by ε_reactive position_ ≈ n/x _*_ ε_bulk_C and AKIE = 1/(1 + z*ε_reactive position_/1,000) where *n* is the number of atoms of interest, *x* the number of atoms located at reactive sites, and *z* the number of indistinguishable atoms at reactive sites.

^5^Calculated for fumarate addition at C2.

^6^Average fractionation factor from incubations with single gases and incubations with mixture of gases.

^7^Calculated in Jaekel et al. (2014), based on the average values reported by Kinnaman et al. (2007).

^8^Calculated for monohydroxylation at C1 or C2.

### 3.2. Comparison to literature data

Generally, previous isotope fractionation data for aerobic ethane, propane and butane degradation have been determined in complex microbial communities (Table 1) where the alkanes might have been simultaneously degraded by different organisms using different enzymes and pathways, hence leading to a mixed isotope fractionation factor representing the average sum of different isotope fractionation factors. In contrast, the isotope fractionation values presented here are the first ones generated for aerobic oxidation of alkanes by a pure culture containing a single enzyme for alkane oxidation and may serve as a standard for future investigations using other model strains harboring different alkane-activating enzymes.

#### 3.2.1. Ethane

The ε_bulk_H value for ethane monooxygenation (−56.3 ‰) is similar to those reported for aerobic ethane biodegradation by undefined microbial communities of marine sediments, whereas the ε_bulk_C ethane observed here for *T. butanivorans* (−2.95 ‰) is significantly smaller than that observed for the same marine microbial community (Table 1; Kinnaman et al., 2007). This resulted in significantly different Λ values of aerobic ethane biodegradation (16 of *T. butanivorans* vs. 8 of the marine community). As pointed out above, the fractionation factors determined for the marine community may be caused by the simultaneous operation of different ethane degradation pathways and cannot be directly compared. The *Λ* window for aerobic methane degradation, a reaction catalyzed by methane monooxygenase and characterized by strong carbon and hydrogen isotope fractionation, is considerably smaller and lies between 7 and 11, determined using several pure cultures representing distinct methane monooxygenases (Feisthauer et al., 2011). No enrichment factors have been determined yet for ethane biodegradation under anoxic conditions, thus it remains to be shown whether aerobic and anaerobic ethane biodegradation can be differentiated by ME-CSIA, and if anaerobic or microaerophilic oxidation may have contributed to the C and H fractionation during ethane oxidation in marine sediments.

#### 3.2.2. Propane

Similar to aerobic ethane degradation, the isotope fractionation pattern of propane monooxygenation by *T. butanivorans* was different from values described before for an aerobic propane-degrading microbial community from marine sediments (Table 1; Kinnaman et al., 2007). Particularly, ε_bulk_H values of the marine community were considerably lower than those observed for *T. butanivorans*, resulting in different *Λ* values (*Λ* = 13 for *T. butanivorans* vs. *Λ* = 3 for the marine community, Table 1). As discussed above, the previously determined fractionation factors for this complex marine community cannot be directly linked to a single enzymatic reaction as for *T. butanivorans*, preventing a direct comparison of these factors. Notably, the obtained values for *T. butanivorans* are in the same range determined for anaerobic activation of propane by a microbial community from marine sediment, marine enrichment cultures and a marine pure strain (Table 1; Mastalerz et al., 2009; Jaekel et al., 2014), the latter two activating propane by fumarate addition. This picture is general complicated by the fact that isotope fractionation has been shown to be significantly affected by mass-transfer limitations in biodegradation experiments, since continuously mixed cultures showed higher isotope fractionation than non-mixed, mass-transfer limited cultures (Jaekel et al., 2014; Table 1).

Nevertheless, the current data indicates that propane biodegradation is generally trackable by ME-CSIA at various environmental conditions due to strong carbon and hydrogen isotope fractionation, but degradation under oxic and anoxic conditions cannot be easily differentiated. A similar picture was observed for analysis of methane biodegradation by ME-CSIA (Feisthauer et al., 2011).

#### 3.2.3. Butane

The ε_Cbulk_ values for aerobic butane biodegradation by soil and marine microbial communities are higher than the value determined here for *T. butanivorans* (Table 1); however, to date no ε_Hbulk_ have been determined for aerobic butane degradation, hence no Λ values can be compared. Bulk carbon and hydrogen fractionations of butane monooxygenation by *T. butanivorans* are in the same range or significantly lower as values determined for anaerobic butane activation by fumarate addition by marine enrichment cultures and pure strains (Table 1; Jaekel et al., 2014). However, the Λ value (11.4 ± 2.8) for *T. butanivorans* is at the upper range observed for anaerobic butane degradation (*Λ* values =4.9 ± 1.2 to 8.7 ± 0.4) (Table 1). This range overlap challenges the distinctions of anaerobic and aerobic butane degradation based on isotope values.

## 4. Conclusions and environmental implications

Our data show that monooxygenation of the short-chain, volatile alkanes ethane, propane and butane is associated with strong carbon and hydrogen stable isotope fractionation, demonstrating the general applicability of ME-CSIA for assessing biodegradation of such alkanes in the environment. Identifying this reaction solely by its compound-specific Λ values is however difficult due to the similarity of Λ values observed for monooxygenation (oxic conditions) and addition to fumarate (anoxic conditions). Similar observations were made for methane oxidation under oxic and anoxic conditions (Feisthauer et al., 2011). The current landscape of C and H fractionation associated with volatile alkane oxidation can be completed by acquiring additional ME-CSIA data of enzyme-specific alkane hydroxylation reactions. While the sBMO of *T. butanivorans* is a non-haem diiron enzyme related to soluble methane monooxygenases, other enzymes initiating the oxidation of volatile alkanes belong to the AlkB family of alkane hydroxylases, an enzyme family with a high sequence diversity (Rojo 2009). Diverse enzymes may cause slightly different reaction mechanisms and may result in different ε_bulkC_, ε_bulkH_ and Λ values as observed here (Table 1). Such effects have been formerly described for other highly diverse classes of enzymes. For example, benzylsuccinate synthases catalyzing the anaerobic activation of toluene show a high sequence diversity, and display a broad range of ε_bulkC_, ε_bulkH_ and Λ values essentially for the same enzymatic reaction (Vogt et al., 2008; Kümmel et al., 2013). The recent discovery of archaea oxidizing volatile alkanes like ethane and butane via alkyl-coenzyme *M* reductases, or ACR (Laso-Perez et al., 2016; Chen et al., 2019; Seitz et al., 2019; Hahn et al., 2020, 2021) adds to the complexity of distinguishing between aerobic and anaerobic processes in environmental samples. Stable isotope fractionation patterns for ACR-dependent pathways have not been reported so far. Judging from ME-CSIA values determined for the analogous pathway of anaerobic oxidation of methane by methyl-coenzyme *M* reductases (Holler et al., 2009), one can anticipate that ACR-dependent reactions are also associated with strong carbon and hydrogen effects.

## Data availability statement

The raw data supporting the conclusions of this article will be made available by the authors, without undue reservation.

## Author contributions

FM, CV, and H-HR designed the experiments. ZS performed the experiments. ZS, FM, and CV analyzed the data. CV drafted the manuscript. FM and H-HR revised the manuscript. All authors contributed to the article and approved the submitted version.
