# Supplementary material for: Carbon and hydrogen stable isotope fractionation due to monooxygenation of short-chain alkanes by butane monooxygenase of Thauera butanivorans Bu-B1211
Source: Front Microbiol. 2023 Sep 25;14:1250308. doi: 10.3389/fmicb.2023.1250308 (PMC10560718; doi:10.3389/fmicb.2023.1250308)
Supplement: Supplementary file 1 [file Data_Sheet_1.PDF]

Supplementary Material

1 Supplementary Figure

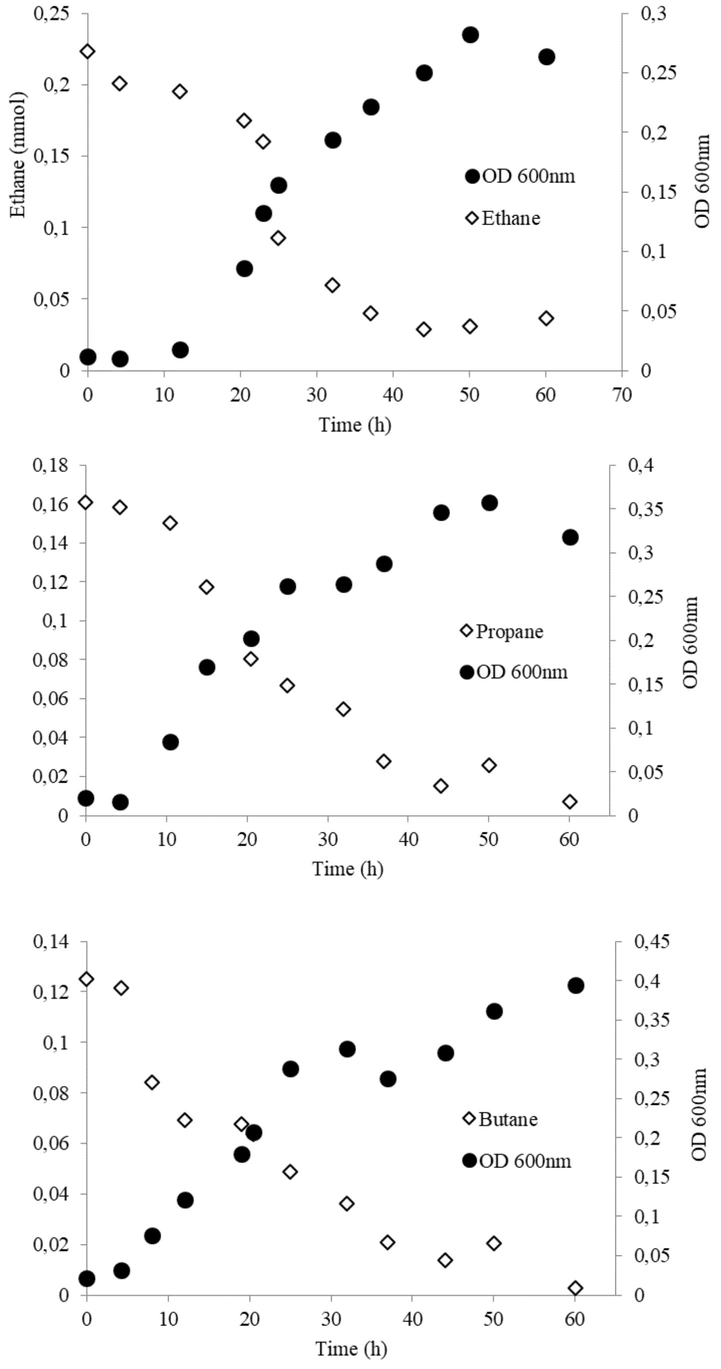

Figure S1. Ethane (A), propane (B), and butane (C) degradation coupled to growth of *T. butanivorans.*
